# Predictors of Poor CD4 and Weight Recovery in HIV-Infected Children Initiating ART in South Africa

**DOI:** 10.1371/journal.pone.0033611

**Published:** 2012-03-16

**Authors:** Brian C. Zanoni, Thuli Phungula, Holly M. Zanoni, Holly France, E. Francis Cook, Margaret E. Feeney

**Affiliations:** 1 The Ragon Institute of MGH, MIT and Harvard, Charlestown, Massachusetts, United States of America; 2 Harvard Medical School, Boston, Massachusetts, United States of America; 3 Sinikithemba Clinic and Philani Program, McCord Hospital, Durban, South Africa; 4 Harvard School of Public Health, Boston, Massachusetts, United States of America; 5 Division of Experimental Medicine, University of California San Francisco, San Francisco, California, United States of America; Mayo Clinic, United States of America

## Abstract

**Objective:**

To identify baseline demographic and clinical risk factors associated with poor CD4 and weight response after initiation of antiretroviral therapy (ART) in a cohort of human immunodeficiency virus (HIV)-infected children in KwaZulu-Natal, South Africa.

**Methods:**

We performed a retrospective cohort study of 674 children initiating antiretroviral therapy at McCord and St. Mary's hospitals in KwaZulu-Natal, South Africa, from August 2003 to December 2008.

We extracted data from paper charts and electronic medical records to assess risk factors associated with CD4 and weight response using logistic regression.

**Results:**

From the initial cohort of 901 children <10 years old initiating ART between August 2003 and December 2008, we analyzed 674 children with complete baseline data. Viral suppression rates (<400 copies/ml) were 84% after six months of therapy and 88% after 12 months of therapy. Seventy-three percent of children achieved CD4 recovery after six months and 89% after 12 months. Weight-for-age Z-score (WAZ) improvements were seen in 58% of children after six months of ART and 64% after 12 months. After six months of ART, lower baseline hemoglobin (p = 0.037), presence of chronic diarrhea (p = 0.007), and virologic failure (p = 0.046) were all associated with poor CD4 recovery by multivariate logistic regression. After 12 months of ART, poor CD4 recovery was associated with higher baseline CD4% (p = 0.005), chronic diarrhea (p = 0.02), and virologic failure (p<0.001). Age less than 3 years at ART initiation (p = 0.0003), higher baseline CD4% (p<0.001), and higher baseline WAZ (p<0.001) were all associated with poor WAZ improvements after 6 months by multivariate logistic regression.

**Conclusion:**

The presence of chronic diarrhea at baseline, independent of nutritional status and viral response, predicts poor CD4 recovery. Age at initiation of ART is an important factor in early WAZ response to ART, while viral suppression strongly predicts CD4 recovery but not WAZ improvement.

## Introduction

Antiretroviral therapy (ART) has been shown to dramatically improve clinical outcomes, immune status, and growth parameters among children infected with HIV [1], [2], [3], [4], [5], [6], [7], [8]. Children's immune and weight response to ART differs based on age at ART initiation and degree of viral suppression [3], [9], [10], [11]. As shown in recent adult studies [12], there are likely other baseline factors contributing to differential immunologic responses. Baseline clinical and demographic factors have been used to predict mortality in HIV-infected children on ART [13], [14], [15], [16], [17]. However, few baseline clinical characteristics other than age, CD4% and viral response have been examined as potential predictors of weight and immune response in HIV-infected children.

Published cohort studies have generated conflicting results regarding baseline CD4% and age as predictors of CD4 recovery [3], [10], [11], [18]. Some studies found improved early CD4 response in younger children [3], [10], [11], while others found no difference [18]. A similar discrepancy exists for baseline CD4% as a predictor of CD4 response [10]. These studies, however, did not assess other potential clinical predictors of early immunologic and weight response such as the presence of chronic diarrhea, tuberculosis, opportunistic infections or anemia. Clinicians could potentially use these factors to identify children at higher risk of poor immune response to ART and target them for more intense monitoring and closer follow-up.

We performed a retrospective analysis of baseline clinical and demographic risk factors as predictors of CD4 recovery and weight gain six and 12 months after ART initiation in a cohort of children from two medical centers in South Africa. Our goal was to identify clinically significant risk factors for poor weight gain and CD4 recovery in children taking ART.

## Materials and Methods

### Ethics Statement

The protocol was approved by McCord Hospital's Research Ethics Committee, St. Mary's Hospital's Ethics Committee and the Partners Human Research Committee. All patients (or their adult guardians/care givers) accessing care at McCord Hospital signed a written consent authorizing storage of their medical information on an electronic medical record database used for clinical and research purposes. We did not obtain individual consent.

### Study Design

We performed a retrospective cohort study using paper charts and electronic medical records from HIV-infected pediatric patients (<10 years old) who initiated ART at McCord Hospital's Sinikithemba Clinic and St. Mary's Hospital in KwaZulu-Natal, South Africa, from August 2003 to December 2008. We analyzed clinical and demographic characteristics at the time of ART initiation, as well as, viral suppression at six and 12 months post-ART initiation as potential predictors of CD4 and WAZ response.

### Study Population and Standard of Care

McCord Hospital is a semi-private, urban hospital providing care for a mostly Zulu-speaking population in Durban, South Africa. St. Mary's Hospital is a semi-private, rural hospital for a mostly Zulu-speaking population in Marrianhill, South Africa. A total of 901 children <10 years old initiated ART at McCord Hospital's Sinikithemba Clinic and St. Mary's Hospital during the study period. We followed patients from the time they initiated ART until they died, transferred care to another facility, were lost to follow-up or until the study end date of May 31, 2009. During the study period, children initiated ART when their HIV disease reached World Health Organization (WHO) stage 3 or 4 and/or their CD4 percentage fell to less than 20% in children younger than 18 months of age, or less than 15% in children older than 18 months of age, in accordance with South African National Treatment Guidelines [19]. Based on national guidelines in South Africa, children less than 3 years of age received a Protease Inhibitor (PI)-based first-line treatment regimen comprised of lopinavir/ritonavir, stavudine and lamivudine [19]. Children older than 3 years of age initiated a Non-Nucleotide Reverse Transcriptase Inhibitor (NNRTI)-based treatment regimen comprised of efaverinez, stavudine and lamivudine [19]. According to local guidelines, routine laboratory monitoring includes baseline CD4 and six monthly CD4 and viral loads [19].

### Data Collection

We evaluated medical records from all patients who were ≤10 years old when they initiated ART at McCord Hospital's Sinikithemba Clinic (August 2003 to December 2008) and St. Mary's Hospital (January 2007 to December 2008). We accessed electronic medical records at McCord Hospital using TrackCare software and cross-referenced these records with paper charts. At St. Mary's Hospital, we evaluated conventional paper medical records and, due to a change in documentation procedures at St. Mary's Hospital, we did not include medical records prior to 2007. We entered the following data into a Microsoft Access database: age at ART initiation, gender, ART regimen, presence of tuberculosis (TB) and non-TB opportunistic infections, chronic diarrhea (longer than 14 days), and baseline laboratory results including: absolute and CD4 percentage, hemoglobin and baseline weight-for-age Z-scores (WAZ) (calculated for all children less than 10 years of age using the WHO macro for STATA; https://www.who.int/childgrowth/software). We recorded the presence of chronic diarrhea, tuberculosis and opportunistic infections based on their presence in the electronic medical record and following a review of the hard copy paper records. We defined opportunistic infections (OI) as the presence of: oral candidiasis, Kaposi's sarcoma, Pneumocystis jiroveci pneumonia, cryptococcosis, toxoplasmosis, or cryptosporidiosis. We treated all OIs according to the South African National Treatment Guidelines for Children [20]. If these conditions were not documented in the electronic medical record or paper charts, we reported them as absent.

#### Missing Data

In multivariate analysis, we only analyzed subjects with complete data. No data was imputed for this analysis.

#### Definition of Poor CD4 Recovery

We defined poor CD4 recovery for children less than 5 years old on therapy for more than 24 weeks as: less than five point increase CD4% or CD4% remaining less than 15%. For children older than 5 years, poor CD4 recovery was defined as: <10% increase in absolute CD4, or absolute CD4 remaining below 200 cells/mm^3^. These definitions were based on the WHO classification of immunological failure for children [21] and adolescents [22] and other reports in the literature [10].

#### Definition of Poor WAZ Response

We defined poor weight gain based on weight-for-age Z-scores (WAZ). We, therefore, limited the analysis to children less than 10 years old because WHO weight standardizations only exist for this age group. Poor WAZ response was defined as: any decrease from pre-ART baseline WAZ after six months of ART, or less than 0.5 point increase in WAZ for children with baseline WAZ<−0.5.

### Statistical Analysis

We conducted statistical analyses using SAS statistical software (Release 9.2, Carey, NC). Using logistic regression, we first determined univariate associations between nine demographic and clinical covariates, which, based upon clinical observations and prior studies, were suspected to be potentially important correlates of poor immunologic or clinical response. The covariates included: age at initiation, sex, baseline absolute CD4, baseline CD4%, baseline hemoglobin, baseline WAZ, presence of TB, presence of OIs, and presence of chronic diarrhea. We stratified age into three categories (<1 year old, 1–3 years old, and 3–10 years old) to account for the age-related difference in ART treatment regimens. For all analyses, we selected children ages 3–10 years as the reference group. In our final model, we used multivariate logistic regression controlling for age at initiation. The final model was selected from the remaining eight covariate model using a backward elimination procedure with p<0.05 considered significant to remain in the model.

We did not assess the association between type of ART regimen (Protease Inhibitor verses Non-Nucleotide Reverse Transcriptase Inhibitor) and CD4 or WAZ response in these analyses because the ART regimen was selected based on age and, thus, these variables were highly correlated (Pearson correlation coefficient (PCC) = 0.79). In addition, absolute CD4 and CD4% were highly correlated (PCC = 0.65); therefore we chose to include only CD4% as a covariate. Chronic diarrhea and OIs; and chronic diarrhea and WAZ were not highly correlated (PCC = 0.13 and −0.16 respectively); therefore, they were included as separate covariates in the final models.

## Results

**Figure 1:**
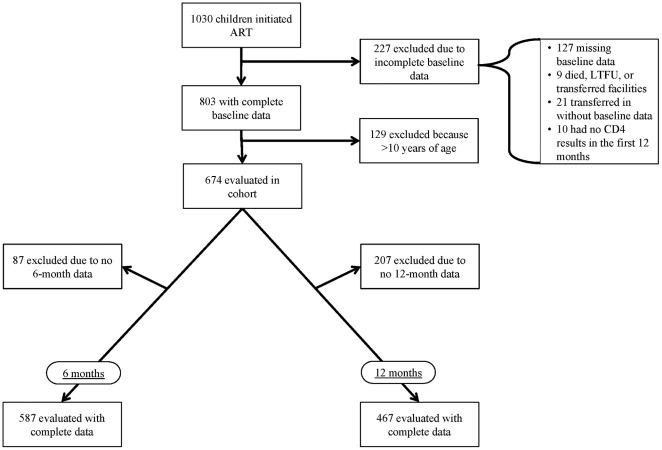
Selection of Children for Analysis from a Cohort of HIV-infected <10 Years Old Children Initiating ART, South Africa, August 2003–December 2008.

A total of 1,030 children initiated ART therapy at McCord Hospital and St. Mary's Hospital between August 2003 and December 2008. Of these, 647 were eligible for the study (Figure 1). Of those who were ineligible: 127 patients were missing baseline CD4 results; 69 children died, were lost to follow up or transferred to another facility within the first six months of ART; 21 children transferred into care without documentation; 10 children did not have CD4 results within the first 12 months of therapy; and 129 children were older than 10 years at ART initiation. Clinical and demographic characteristics for this cohort of children <10 years old are located in Table 1. After six months of ART, 587 had complete results. After 12 months of ART, 467 children had complete results. Due to the amount of missing and unavailable data, we evaluated characteristics of those children with and without available follow-up results (Table 1). At six months, there was no statistically significant difference between those children with available results and those with unavailable results. At 12 months, those with available results had higher baseline absolute CD4 and had a higher rate of tuberculosis at ART initiation.

**Table 1:** Baseline Clinical and Demographic Characteristics of a Cohort of HIV-infected <10 Years Old Children Initiating ART, by CD4 Availability at Six and 12 months, South Africa, August 2003–December 2008.

	6 Months			12 Months		
Covariate	Results Available N = 587	Results Not Available N = 87	P-value	Results Available N = 467	Results Not Available N = 207	P-value
**Median Age at Initiation (years)[IQR]**	4.0 [1.4–6.8]	4.0 [1.1–7.4]	0.94	4.1 [1.5–6.8]	3.6 [1.3–6.9]	0.62
**Females**	48%	44%	0.49	45%	53%	0.54
**Baseline CD4 (Median) (cells/µL) [IQR]**	364 [143–686]	364 [157–723]	0.90	358 [133–665]	380 [196–754]	0.09
**Baseline CD4 Percent (Median) [IQR]**	12 [6.6–16.3]	12 [6]–[19]	0.58	12 [6]–[15]	13 [8]–[19]	0.001
**Baseline Hemoglobin (Median) (g/dl) [IQR]**	9.8 [8.6–10.7]	10.0 [9.1–10.6]	0.31	9.9 [8.7–10.7]	9.6 [8.4–10.7]	0.23
**NNRTI** *	61.5%	62.1%	1	63.2%	58.0%	0.23
**TB Co-infection** †	37.3%	28.7%	0.15	33.0%	43.5%	0.01
**Chronic Diarrhea**	14.1%	12.9%	0.87	14.6%	12.6%	0.55
**Baseline Weight for Age Z-score (Median) [IQR]**	−1.2 [−2.4–−0.4]	−1.3 [−2.3–−0.1]	0.72	−1.3 [−2.5–0.4]	−1.1 [−2.3–−0.4]	0.84
**Opportunistic Infection** ‡	23.8%	21.8%	0.79	24.6%	21.3%	0.38

*Children less than 3 years are started on a PI-based regimen while children over 3 years are started on an NNRTI-based regimen.

† Baseline tuberculosis co-infection.

‡ *Opportunistic infection* includes children with the presence of candidiasis, Kaposi's sarcoma, Pneumocystis jiroveci pneumonia, cryptococcosis, toxoplasmosis, or cryptosporidiosis.

**Figure 2:**
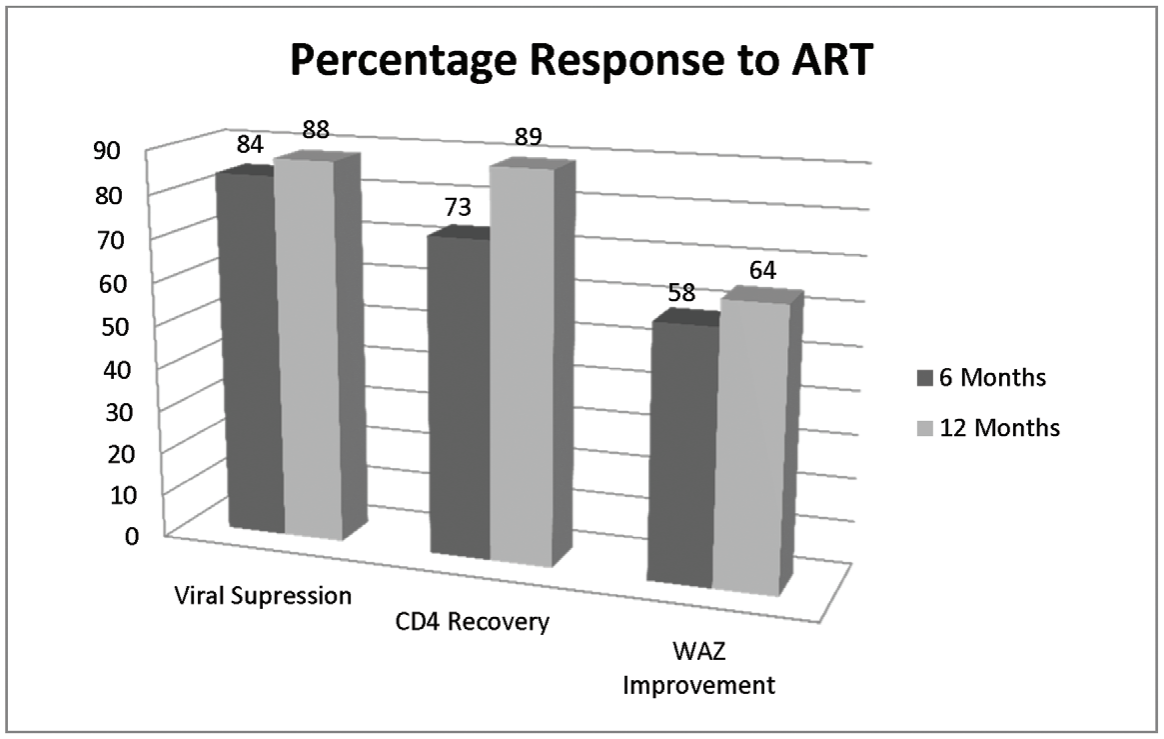
Percentage Response to ART at Six and 12 Months for a Cohort of HIV-infected <10 Years Old Children Initiating ART, South Africa, August 2003–December 2008.

Overall response to ART is shown in Figure 2. Viral suppression rates (to less than 400 copies/ml) were 84% after six months of therapy and 88% after 12 months of therapy. CD4 recovery (as defined above) was achieved in 73% of children after six months and 89% after 12 months. After six months of ART, 58% of children had improved WAZ (defined above), and 64% of children achieved an improved WAZ after 12 months. Mortality in this cohort was 8.8%. There was no difference in viral suppression, immunologic response or weight response based on location of care.

### Predictors of CD4 Recovery

We performed univariate logistic regression (Table 2) and multivariate logistic regression (Table 3) to identify predictors of CD4 response to ART after six and 12 months of therapy. The final multivariate logistic regression model for the six-month analysis revealed that presence of chronic diarrhea (p = 0.007), lower baseline hemoglobin (p = 0.037) and virologic failure (p = 0.046) were significantly associated with poor CD4 recovery. In multivariate analysis, children with documented chronic diarrhea at baseline were more likely to have poor CD4 recovery (OR = 2.05; 95% CI 1.22–3.46) independent of baseline nutritional status. Children with lower baseline hemoglobin had significantly worse CD4 recovery (OR = 0.87 for each 1 g/dl decrease in hemoglobin; 95% CI 0.75–0.99). Additionally, children whose viral load remained >400 copies/ml at six months were more likely to have poor CD4 recovery at six months (OR = 1.75; 95% CI: 1.01–3.03).

**Table 2:** Univariate Predictors of Poor CD4 Response From a Cohort of HIV-infected <10 Years Old Children Initiating ART in South Africa, August 2003–December 2008.

	6 Months N = 587		12 Months N = 467	
	Odds Ratio [95% CI]	P-value	Odds Ratio [95% CI]	P-value
**Age at Initiation** *		0.005		0.26
**Age <1 year**	2.1 [1.4–3.4]	0.001	1.54 [0.80–2.96]	0.19
**Age 1–3 years**	1.4 [0.9–2.2]	0.14	0.80 [0.29–1.65]	0.54
**Females**	0.93 [0.97–1.02]	0.68	1.15 [0.67–1.98]	0.61
**Baseline CD4 %**	1 [0.97–1.02]	0.98	1.07 [1.03–1.11]	0.0002
**Baseline Hemoglobin**	0.81 [0.71–0.92]	0.001	1.02 [0.89–1.19]	0.77
**TB Co-infection**	1.15 [0.79–1.68]	0.46	0.93 [0.52–1.67]	0.82
**Chronic Diarrhea**	1.85 [1.14–2.99]	0.01	1.78 [0.90–3.48]	0.09
**Baseline WAZ**	0.99 [0.89–1.10]	0.80	0.99 [0.84–1.19]	0.98
**Opportunistic Infection** †	1.53 [1.02–2.31]	0.04	0.83 [0.43–1.59]	0.57
**Viral Failure**	2.08 [1.20–3.34]	0.003	2.03 [0.96–4.30]	0.06

*Age was treated as a categorical variable including age <1 year, 1–3 years, 3–10 years, where children 3–10 were the reference group.

† *Opportunistic infection* includes children with the presence of candidiasis, Kaposi's sarcoma, Pneumocystis jiroveci pneumonia, cryptococcosis, toxoplasmosis, or cryptosporidiosis, while the reference group did not have any of these opportunistic infections.

**Table 3:** Multivariate Predictors of Poor CD4 Response From a Cohort of HIV-infected <10 Years Old Children Initiating ART in South Africa, August 2003–December 2008.

	6 Months† N = 587		12 Months‡ N = 476	
	Odds Ratio [95% CI]	P-value	Odds Ratio [95% CI]	P-value
**Age at Initiation** *		0.13		0.99
**Baseline Cd4%**			1.07 [1.02–1.13]	0.005
**Baseline Hemoglobin**	0.87 [0.75–0.99]	0.037		
**Chronic Diarrhea**	2.05 [1.22–3.46]	0.007	2.58 [1.15–5.79]	0.02
**Viral Failure**	1.75 [1.01–3.03]	0.046	4.01 [1.77–9.07]	0.0009

*Age was treated as a categorical variable including age <1 year, 1–3 years, 3–10 years, where children 3–10 were the reference group.

† In the 6 month multivariate logistic regression model age at initiation was forced in and baseline hemoglobin, chronic diarrhea and viral failure were selected by backward elimination procedure with p<0.05 as criteria for inclusion in the model.

‡ In the 12 month multivariate logistic regression model age at initiation was forced in and baseline CD4%, chronic diarrhea and viral failure were selected by backward elimination procedure with p<0.05 as criteria for inclusion in the model.

After 12 months on ART, multivariate logistic regression revealed that higher baseline CD4%, presence of baseline chronic diarrhea and virologic failure were significantly associated with poor CD4 recovery (p = 0.005; p = 0.02; p<0.001, respectively) controlling for age at initiation. Children with chronic diarrhea at baseline were more likely to have poor CD4 recovery at 12 months (OR = 2.58; 95% CI: 1.15–5.79). Virologic failure at 12 months was the strongest predictor of CD4 response at 12 months. Children who did not have suppressed viral loads after 12 months of ART were more likely to have poor CD4 recovery (OR = 4.01; 95% CI: 1.77–9.07).

### Predictors of WAZ Improvement

Next, we performed univariate logistic regression (Table 4) and multivariate logistic regression (Table 5) to evaluate weight gain following ART initiation. The final multivariate logistic regression model for the six-month analysis revealed that age at initiation (p<0.001), baseline CD4% (p<0.001) and baseline WAZ (p<0.001) were significantly associated with weight response. Children aged 3–10 years at the time of ART initiation were more likely to experience a poor weight response compared to children aged 1–3 years at initiation (p = 0.008; 95% CI: 0.31–0.84) and those less than 1 year old (p<0.001; 95% CI 0.17–0.58). Children with lower baseline WAZ had improved WAZ compared to children with a higher baseline WAZ. There was a 44% increase in adequate WAZ response for every one point lower baseline WAZ (OR = 1.44; 95% CI: 1.26–1.65). After 12 months on ART, baseline WAZ was the only variable significantly associated with adequate WAZ response (p<0.0001) controlling for age at initiation.

**Table 4:** Univariate Predictors of Poor WAZ Response From a Cohort of HIV-infected <10 Years Old Children Initiating ART in South Africa, August 2003–December 2008.

	6 Months N = 587		12 Months N = 476	
	Odds Ratio [95% CI]	P-value	Odds Ratio [95% CI]	P-value
**Age at initiation** *		0.005		0.04
**Age <1 year**	0.47 [0.39–0.77]	0.003	0.60 [0.35–1.02]	0.058
**Age 1–3 years**	0.64 [0.42–0.99]	0.047	0.58 [0.35–0.97]	0.037
**Females**	1.21 [0.85–1.72]	0.29	1.23 [0.82–1.85]	0.31
**Baseline CD4 %**	1.05 [1.02–1.07]	0.0005	1.01 [0.98–1.03]	0.76
**Baseline Hemoglobin**	1.16 [1.03–1.31]	0.016	1.21 [1.06–1.39]	0.006
**TB Co-infection**	0.70 [0.49–1.01]	0.056	0.94 [0.61–1.44]	0.77
**Chronic Diarrhea**	0.54 [0.33–0.91]	0.021	0.69 [0.39–1.22]	0.20
**Baseline WAZ**	1.47 [1.30–1.67]	<0.0001	1.53 [1.33–1.77]	<0.0001
**Opportunistic Infection**	0.67 [0.44–1.02]	0.06	0.54 [0.33–0.89]	0.015
**Viral Failure**	0.54 [0.32–0.92]	0.02	0.67 [0.36–1.25]	0.21

*Age was treated as a categorical variable including age <1 year, 1–3 years, 3–10 years, where children age 3–10 years were used as the reference group.

**Table 5:** Multivariate Predictors of Poor WAZ Response From a Cohort of HIV-infected <10 Years Old Children Initiating ART in South Africa, August 2003–December 2008.

	6 Months† N = 587		12 Months‡ N = 476	
	Odds Ratio [95% CI]	P-value	Odds Ratio [95% CI]	P-value
**Age at Initiation** *		0.0003		0.18
**Age <1**	0.32 [0.17–0.58]	0.0002	0.72 [0.39–1.33]	0.29
**Age 1–3**	0.51 [0.31–0.84]	0.008	0.59 [0.33–1.07]	0.08
**Baseline CD4%**	1.07 [1.03–1.10]	<0.0001		
**Baseline WAZ**	1.44 [1.26–1.65]	<0.0001	1.60 [1.37–1.88]	<0.0001

*Age was treated as a categorical variable including age <1 year, 1–3 years, 3–10 years with children 3–10 years serving as the reference group.

† In the 6 month multivariate logistic regression model age at initiation was forced in and baseline CD4% and baseline WAZ were selected by backward elimination procedure with p<0.05 as criteria for inclusion in the model.

‡ In the 12 month multivariate logistic regression model age at initiation was forced in and baseline WAZ, was selected by backward elimination procedure with p<0.05 as criteria for inclusion in the model.

## Discussion

The increased availability and earlier initiation of ART in HIV-infected children in sub-Saharan Africa has led to dramatic improvement in the care for HIV-infected children. However, the response to therapy is variable, and many children exhibit poor immunologic response to therapy that may increase their risk of morbidity and mortality despite ART. In this cohort of children <10 years old at ART initiation, we observed that the presence of chronic diarrhea at baseline and virologic failure predicted poorer CD4 outcomes at six and 12 months post-ART initiation. These data may be useful for identifying children at higher risk of poor outcomes who would require closer clinical monitoring.

Our data indicate that the presence of chronic diarrhea at baseline, independent of nutritional status, predicts poor CD4 recovery. Chronic diarrhea has been described as a risk factor for early mortality in HIV-infected children [17] and adults [23], [24] but has not been previously associated with poor CD4 recovery. Since children who died prior to six months of ART were excluded from this analysis, the effect of chronic diarrhea on CD4 recovery in HIV-infected children initiating ART may be understated. The mechanism by which baseline chronic diarrhea leads to poor CD4 recovery in children remains unclear.

Malabsorption of ART could theoretically play a role in slower CD4 recovery; however, the observed association was independent of virologic response to therapy. Although children with chronic enteropathy have been found to have increased mucosal permeability, local expression of inflammatory cytokines, and greater T-cell activation [25], [26], [27], it is not known whether or by what mechanism this localized GI inflammation contributes to delayed systemic CD4 recovery in HIV-infected children. Whether the presence of chronic diarrhea in HIV-infected children reflects the gut enteropathy that immediately follows HIV-1 infection warrants further investigation [28], [29].

Adults with immunologic failure have been found to have increased markers of microbial translocation and immune activation; [12]. This previously has not been linked to baseline presence of chronic diarrhea. However, bacterial products have been shown to induce a permanent inflammatory state in the GI tract, which potentiates HIV replication and the associated T-cell depletion in the gut-associated lymphoid tissue (GALT) [30]. In addition, the rapid depletion of CD4 T cells from GALT during acute infection and the delayed recovery of GALT CD4 T cells after initiation of ART during chronic infection [31], could influence the interaction between chronic diarrhea and poor peripheral recovery of CD4 T cells. The poor CD4 response to ART among HIV-infected children with chronic diarrhea warrants further mechanistic investigation that could lead to potential therapeutic interventions.

Our data are in agreement with other pediatric studies suggesting that viral suppression is highly predictive of CD4 recovery [9], [11]. Our data suggest that viral suppression is the strongest predictor of CD4 recovery by 12 months of therapy. This finding emphasizes the overall importance of virologic monitoring. Moreover, it suggests that significant cost savings may be achieved in resource-limited settings by limiting CD4 monitoring to children with documented virologic failure. In this cohort, if CD4 monitoring was performed after six months of therapy only for those with virologic failure, it would have led to a cost savings of 110,000 Rand (approximately $15,715 US Dollars) in one year. Fewer than 10% of children in our cohort who achieved virologic suppression at 12 months of ART had a poor immunologic response. Conversely, only 8% of children with virologic failure at 12 months had an adequate CD4 response.

Children less than three years old at initiation, those with lower baseline CD4%, and those with lower baseline WAZ more frequently showed improvement in WAZ after six months of ART. After 12 months of ART, we observed that children with lower baseline WAZ had better improvement in WAZ. These findings suggest a more robust response to weight gain in malnourished children initiating ART compared to non-malnourished children, as seen in other African pediatric HIV cohorts [1], [32]. In addition, we observed a greater weight response to ART in children with greater baseline immunosuppression. This effect is consistent with findings from other pediatric cohorts [2], [33]. In our cohort, viral suppression did not predict weight gain. However, a cohort from Spain found children with virologic failure had worse clinical outcomes and poor weight gain compared to those with viral suppression [5]. However, in the Spanish cohort, the children had a higher baseline WAZ (−0.45) compared to our cohort (−1.3), allowing for less change in WAZ from baseline to within normal limits.

There are several important limitations to this study. Because this was a retrospective study, observational data, including tuberculosis infections, opportunistic infections and chronic diarrhea, may have been unrecorded, potentially biasing our analyses toward the null hypothesis. Many subjects had missing data and were excluded from the analysis. While no difference was seen in clinical or demographic characteristics between those with missing information and those analyzed at six months, those with unavailable results at 12 months had a slightly higher CD4 percentage and a higher rate of tuberculosis, which could limit the generalizability of the 12-month data. We based our definitions of poor CD4 recovery on clinical and WHO-based guidelines for immunological failure [21], [22]. Likewise, we based our definition of poor weight gain on prior reports from the literature [10]. However, there is no clear consensus definition for these outcomes in children. Finally, we were unable to record several potentially significant confounders for the WAZ response outcome, including family income and food insecurity. Analysis of McCord's urban population (who pay user fees) and St. Mary's rural population (who do not pay user fees) showed no difference in viral suppression rates, CD4 response or WAZ response.

Because South African ART guidelines mandate NNRTI-based ART for children initiating therapy after 3 years of age and PI-based therapy for younger children, age and ART regimen are inextricably linked in our cohort. Therefore, any observed age-related difference between children less than 3 years old and those older than 3 years old could be confounded by a differential response to PI- and NNRTI-based treatment regimens. PI-based regimens produce a more rapid increase in CD4 recovery compared to NNRTI-based regimens in adults after 12 weeks of ART [34]. However, there was no difference in CD4 response based on age in our cohort of children <10 years old.

It seems prudent that clinician should closely monitor children less than 1 year old at ART initiation, those with chronic diarrhea at baseline, low baseline WAZ and virologic failure due to an increased risk for poor immune response and/or poor weight gain. Increased access to treatment and early ART initiation will hopefully decrease the amount of chronic diarrhea seen in this pediatric population. Understanding this mechanism of poor CD4 recovery and the interaction between chronic diarrhea, immune activation, microbial translocation and GALT CD4 depletion may lead to adjunctive therapies in the treatment of pediatric HIV infection.
